# Global Research Trends in the Detection and Diagnosis of Dental Caries: A Bibliometric Analysis

**DOI:** 10.1016/j.identj.2024.08.010

**Published:** 2024-09-04

**Authors:** Jane Ching-Lam Lui, Walter Yu-Hang Lam, Chun-Hung Chu, Ollie Yiru Yu

**Affiliations:** Faculty of Dentistry, The University of Hong Kong, Hong Kong, S.A.R., China

**Keywords:** Dental caries, Caries detection, Early diagnosis, Radiography, Fluorescence, Artificial intelligence

## Abstract

This study aims to provide an overview of the global research trends in the detection and diagnosis of dental caries in the past 20 years. A literature search was conducted in the Scopus Database to retrieve studies on the diagnostic approaches for dental caries published from January 2003 to December 2023. The diagnostic approaches in the retrieved studies were examined and the studies were categorized according to the diagnostic approaches investigated. Bibliometric data including journals, countries, affiliations, authors, and numbers of citations of the publications were summarised. The publications’ keyword co-occurrence was analysed using VOSviewer. This bibliometric analysis included 1879 publications investigating seven categories of caries diagnostic approaches, including visual and/or tactile (*n* = 459; 19%), radiation-based (*n* = 662; 27%), light-based (*n* = 771; 32%), ultrasound-based (*n* = 28; 1%), electric-based (*n* = 51; 2%), molecular-based (*n* = 196; 8%) diagnostic approaches, as well as AI-based diagnostic interpretation aids (*n* = 265; 11%). An increase in the annual number of publications on caries diagnostic approaches was observed in the past 20 years. Caries Research (*n* = 103) presented the highest number of publications on caries diagnostic approaches. The country with the highest number of publications was the United States (*n* = 1092). The University of São Paulo was the institution that published the highest number of articles (*n* = 195). The publication with the highest citation has been cited 932 times. VOS viewer revealed that the most frequently occurring keywords were ‘Deep Learning’, ‘Artificial Intelligence’, ‘Laser Fluorescence’ and ‘Radiography’. This bibliometric analysis highlighted an emerging global research trend in the detection and diagnosis approaches for dental caries in the past 20 years. An evident increase in publications on molecular-based caries diagnostic approaches and AI-based diagnostic interpretation aids was perceived over the last 5 years.

## Introduction

Dental caries, also known as tooth decay, is the most prevalent oral health issue affecting people from all over the world. Dental caries is a disease characterized by the dynamic and multifactorial interaction between acid-producing bacteria, fermentable carbohydrates and other host factors including saliva and teeth.^1^ Accumulation of dental plaque, demineralization and destruction of dental hard tissues are manifestations of dental caries.^2^^,^^3^ If left untreated, dental caries can exacerbate into other adverse complications such as pain, infection, tooth loss, masticatory dysfunction and even death.^4^

FDI World Federation has placed an intensifying emphasis on the role of caries detection and diagnosis in the principle of minimal intervention dentistry (MID) for caries management. MID includes early detection and risk assessment of dental caries, remineralisation of demineralised dental hard tissue, prevention of caries in sound teeth, customized dental recalls, minimally invasive operative interventions and repair of defective restorations.^5^ Caries detection and diagnosis is an act of identifying caries from its signs and symptoms.^6^ It forms the basis of subsequent clinical decision-making on disease identification, preventive interventions and treatments. When formulating the treatment plan, the stage and activity of dental caries dictate the management strategies to be undertaken.^7^ Generally, dental caries that are confined to an early stage are recommended to be mitigated by preventive or non-restorative strategies to stop the progression and reverse the demineralisation of carious tissues.^8^^,^^9^ When carious lesions have advanced to moderate or severe stages, minimally invasive operative management with caries control strategies should be implemented.^10^^,^^11^ Therefore, timely and accurate diagnosis of dental caries is the premise of appropriate treatment plans for caries management and proper interventions to arrest the progression of dental caries.^12^

However, it is ubiquitous to have variations among dentists in the clinical diagnosis of a caries lesion in dental practice.^13^ It is also challenging to get an accurate result for caries diagnosis. Due to the non-specificity of the appearance of defects on enamel surfaces, it has always been a dilemma to identify and differentiate caries lesions on the surfaces of the teeth. In addition, carious lesions may be masked by saliva or dental plaque, or located in areas that are difficult to access, heightening the difficulty of caries detection for clinicians.^14^ Compromised conditions for clinical oral examinations, such as incorrect lighting, and confounding effects of post-eruptive changes, such as attrition or traumatic loss of tooth structure may also impair caries detection results.^14^ Taking into account these drawbacks in caries detection and diagnosis, a number of diagnostic approaches have been developed to facilitate clinical caries diagnosis and enhance the accuracy of the diagnostic results, thus defining the best care pathway for patients.

Choosing an appropriate caries diagnostic approach that is individualized and patient-centred results in an optimal health outcome for the patients.^15^ A proper diagnostic approach for detecting and monitoring carious lesions should be valid and reliable, coupled with high sensitivity and specificity.^16^ As current clinical caries diagnostic approaches pose limitations and cannot be applied to all clinical scenarios, clinicians and researchers have been improving the currently available methods and seeking novel methods for caries detection and diagnosis.^16^^,^^17^ Therefore, this bibliometric study aims to provide a holistic overview of the global research trend on the detection and diagnosis for dental caries in the past 20 years.

## Methods

**Fig. 1:**
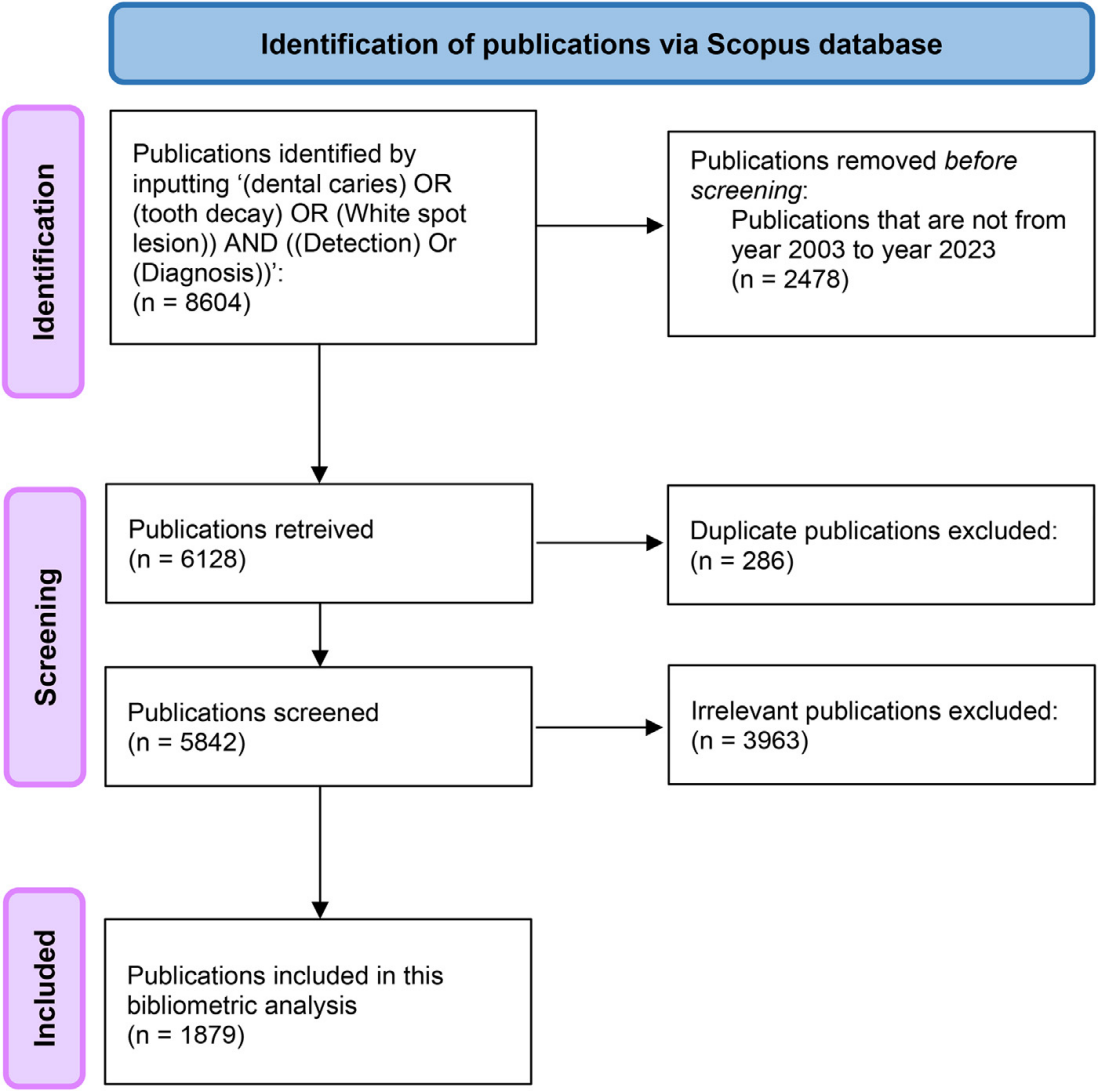
Flowchart of the literature search.

Two independent researchers (JL and OY) conducted a comprehensive literature search in the Scopus database to obtain publications related to the diagnostic approaches for dental caries from January 1, 2003 to December 31, 2023. The keywords used for the literature search were ‘([dental caries] OR [tooth decay] OR [white spot lesion]) AND ([detection] OR [diagnosis])’. Duplicate and irrelevant publications were removed. The keywords, titles and abstracts of the included publications were screened. These publications were then classified according to the diagnostic approaches for caries detection based on their energy sources for further analysis. The flowchart of the literature search is presented in Figure 1.

Bibliometric data including the overall publications on caries diagnostic approaches per year from 2003 to 2023 and the number of publications per caries diagnostic approaches from 2003 to 2023 were examined. These data were extracted from Scopus, inputted on a Microsoft Excel spreadsheet and presented through bar graphs or pie charts. The specific number of publications on each caries diagnostic approach per year from 2003 to 2023 were analysed to scrutinize the current research trend on the diagnostic approaches for dental caries throughout these 20 years. Other bibliometric data such as journals, countries and affiliations with the highest number of publications on caries diagnostic approaches from 2003 to 2023, as well as publications on caries diagnostic approaches with the highest citation counts, were summarized. These data were drawn out from the Scopus database and then inputted into Microsoft Excel to delineate the results.

The keyword co-occurrence of the included publications was analysed using the VOSviewer software (version 1.6.19, Centre for Science and Technology Studies of Leiden University, Netherlands). The keyword co-occurrence refers to the number of publications in which two terms occur together. By inputting the data, a network of keywords was constructed and connected by co-occurrence links. A map with different clusters of items, with each item represented by a circle and a label, was generated according to the network data. The association strength of the co-occurrence links was also displayed by the lines that connect between the items. The data was explored and visualized using network visualization. The bigger the labels, the more prominent and important the items are. Irrelevant keywords such as ‘children’ were eliminated.

## Results

**Fig. 2:**
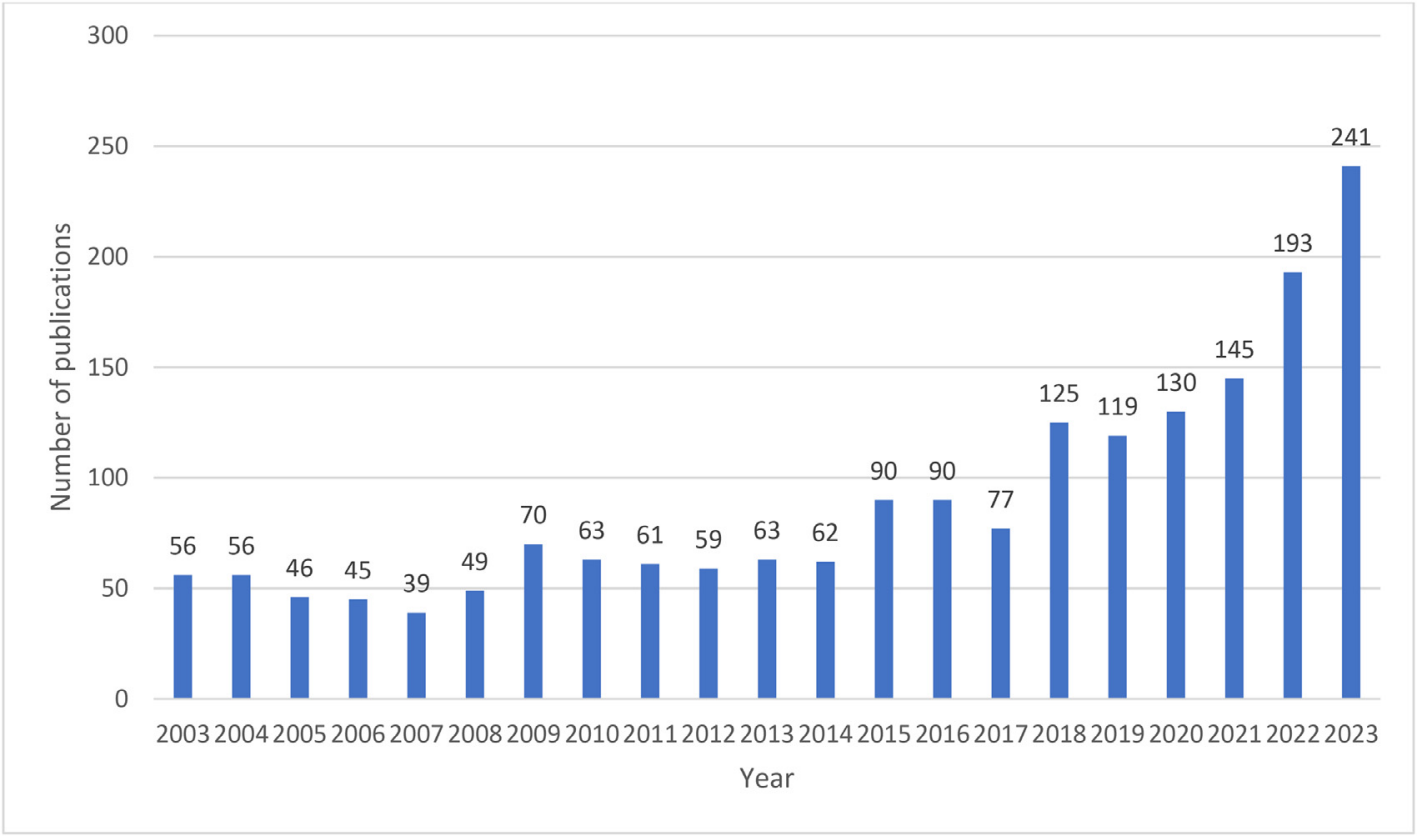
The number of publications on caries diagnostic approaches per year from 2003 to 2023.

This bibliometric analysis included 1879 publications investigating caries diagnostic approaches. The annual number of publications on caries diagnostic approaches from 2003 to 2023 is shown in Figure 2. An increasing trend in the number of publications was observed, from 56 publications in 2003 to 241 publications in 2023. In particular, there was a substantial increase from 2021 to 2023.

**Fig. 3:**
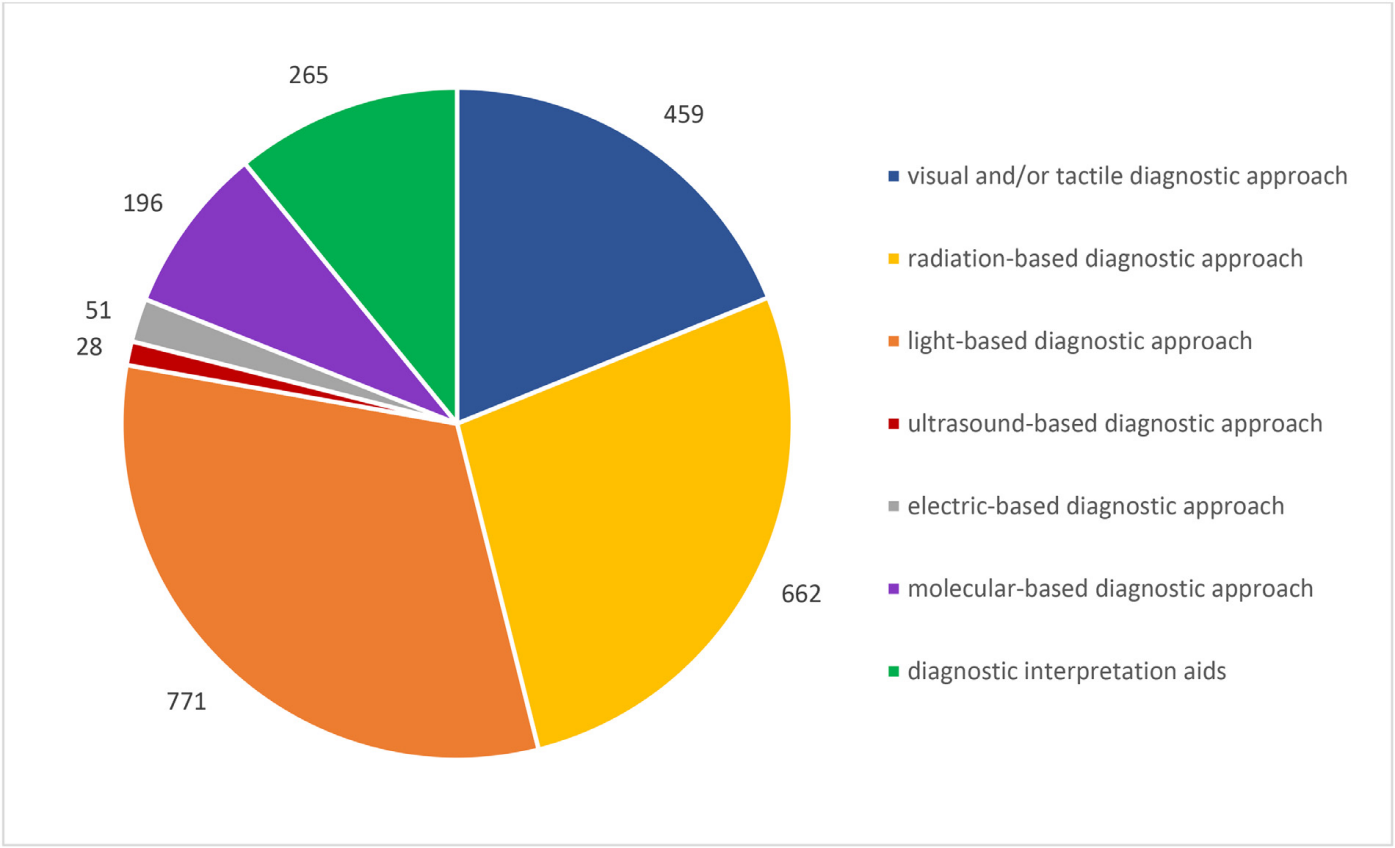
The number of publications per caries diagnostic approach from 2003 to 2023.

These publications were broadly categorized into 7 groups according to the different energy sources of caries detection methods or types of caries diagnostic approaches investigated,^18^ including visual and/or tactile (*n* = 459; 19%), radiation-based (*n* = 662; 27%), light-based (*n* = 771; 32%), ultrasound-based (*n* = 28; 1%), electric-based (*n* = 51; 2%), molecular-based (*n* = 196; 8%) diagnostic approaches, as well as artificial intelligence (AI)-based diagnostic interpretation aids (*n* = 265; 11%) (Figure 3).

**Fig. 4:**
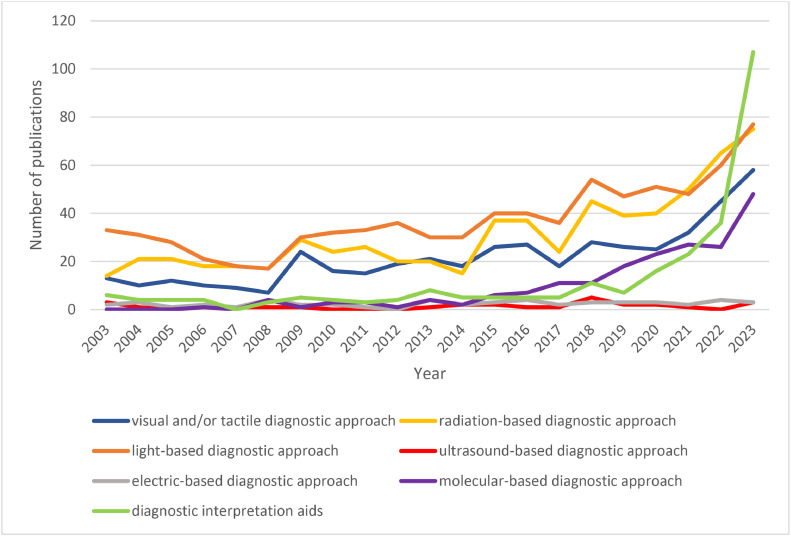
The number of publications on each caries diagnostic approach per year from 2003 to 2023.

Figure 4 revealed an increase in the number of publications related to the visual and/or tactile, radiation-based, and light-based caries diagnostic approaches from 2003 to 2023, reaching a peak in 2023 with 58, 75 and 77 publications, respectively. Publications on ultrasound-based and electric-based caries diagnostic approaches have remained relatively stable throughout the past 20 years, with less than 5 publications per year. On the contrary, molecular-based caries diagnostic approaches and diagnostic interpretation aids have evolved in recent years. There was a remarkable increase from 7 to 48 publications (7-fold increase) in molecular-based caries diagnostic approaches such as saliva biomarkers within 2016-2023. A stark increase was unveiled from 5 to 107 publications in diagnostic interpretation aids such as artificial intelligence within 2016-2023 (21-fold increase).

**Fig. 5:**
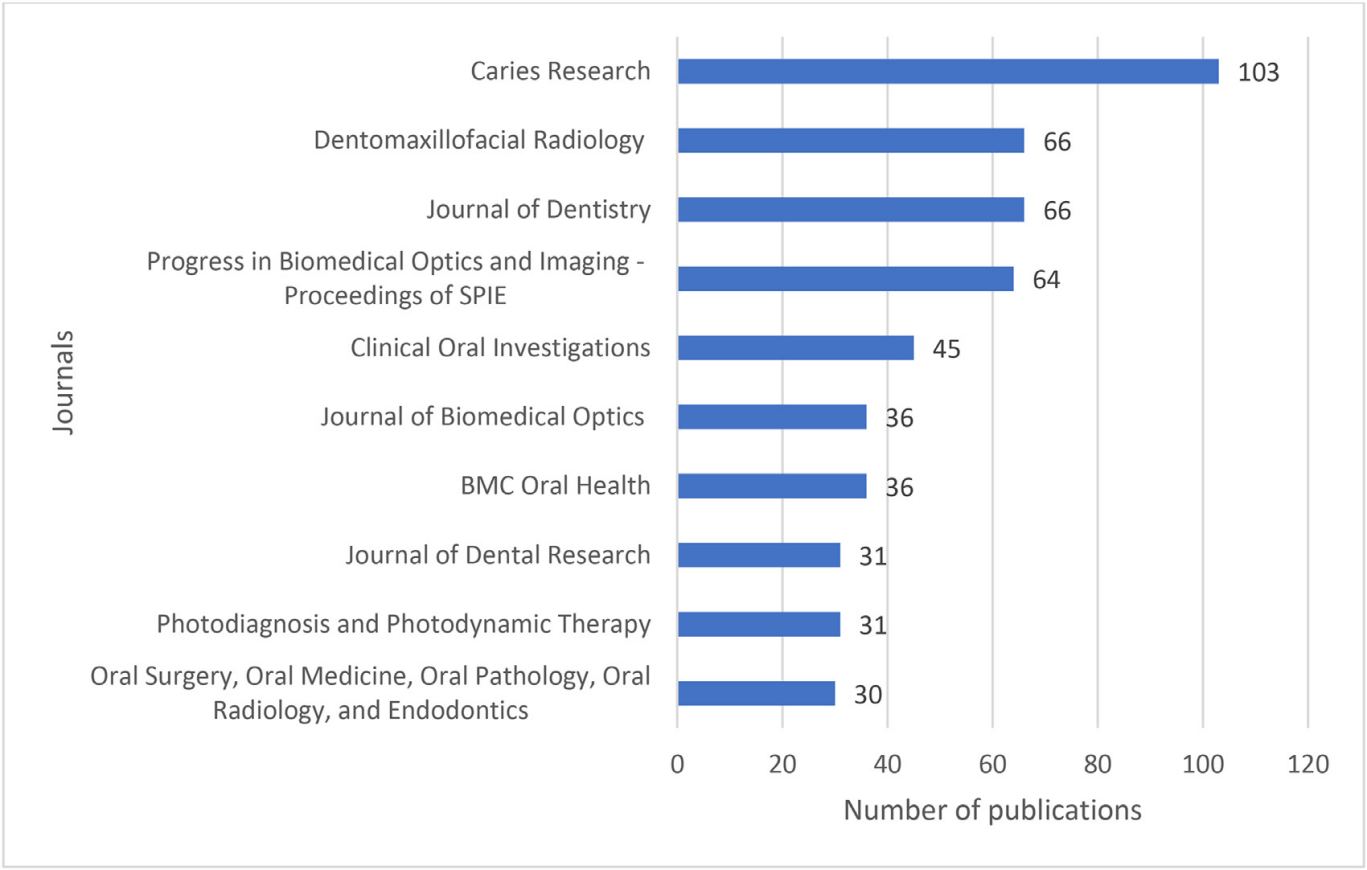
The top 10 journals with the highest number of publications on caries diagnostic approaches from 2003 to 2023.

**Fig. 6:**
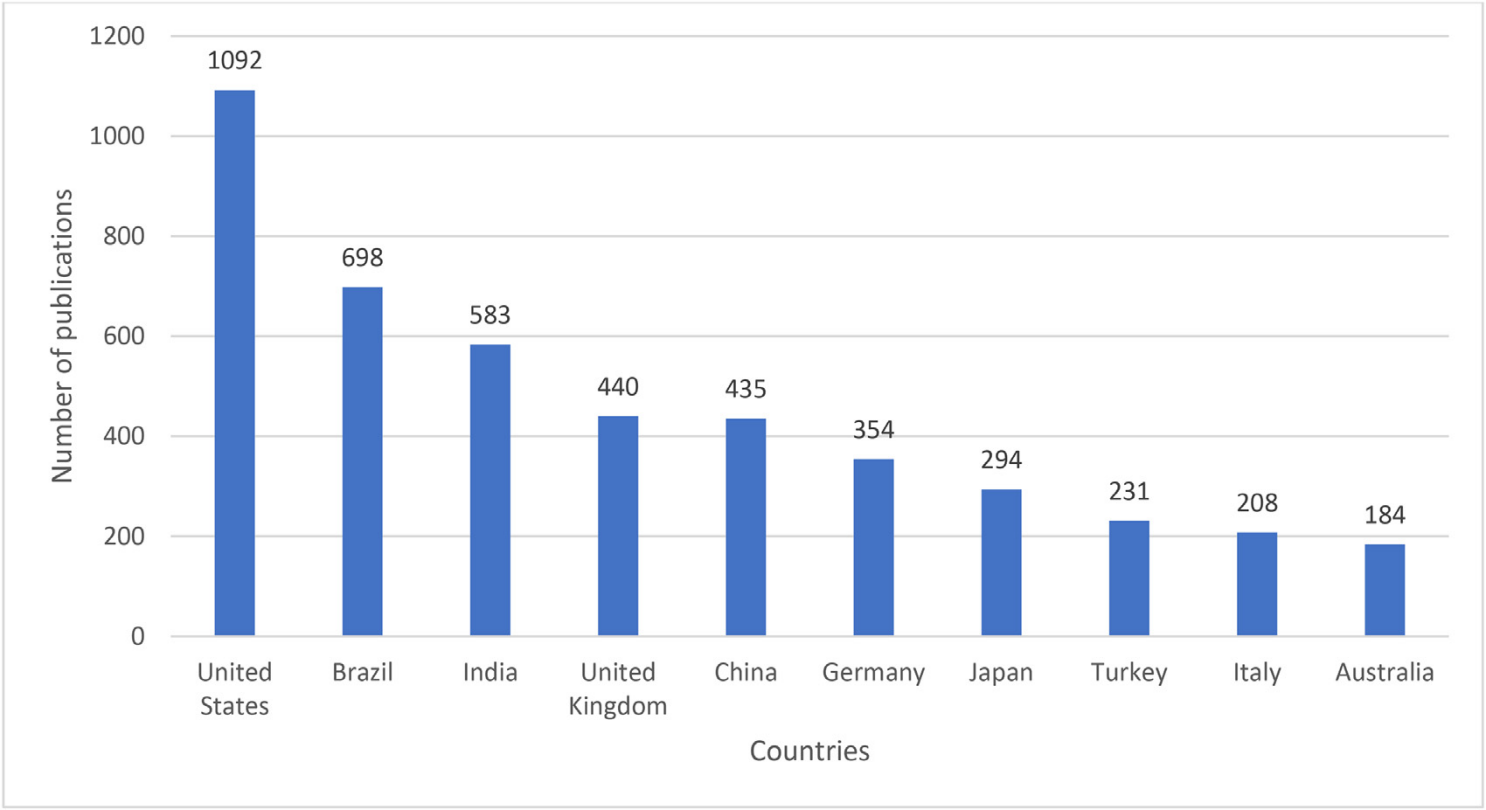
The top 10 countries with the highest number of publications on caries diagnostic approaches from 2003 to 2023.

**Fig. 7:**
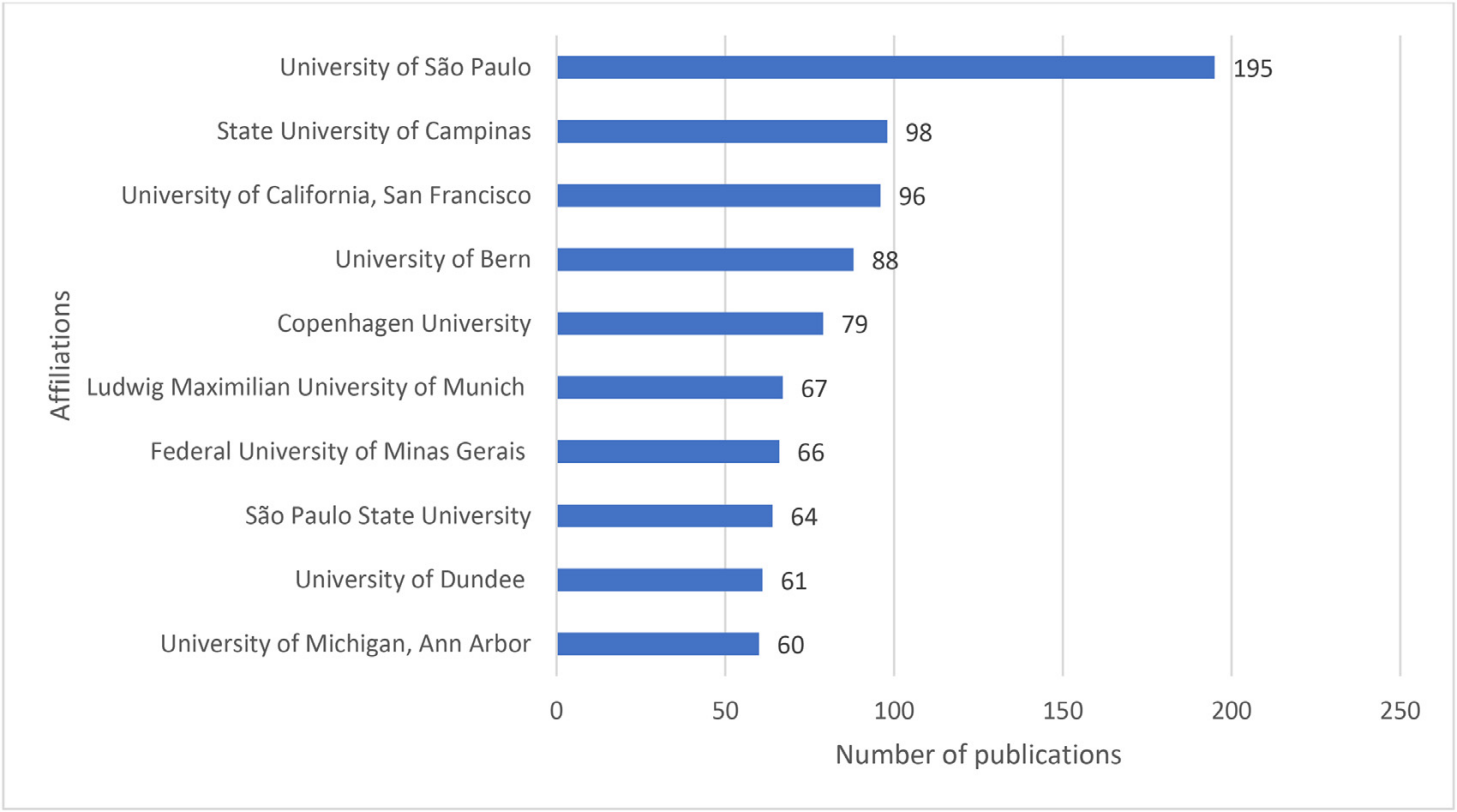
The top 10 affiliations with the highest number of publications on caries diagnostic approaches from 2003 to 2023.

Publications on caries diagnostic approaches were published in different journals by various research teams and affiliations from different countries. Caries Research (*n* = 103), Dentomaxillofacial Radiology (*n* = 66), Journal of Dentistry (*n* = 66), Progress in Biomedical Optics and Imaging-Proceedings of SPIE (*n* = 64), Clinical Oral Investigations (*n* = 45), Journal of Biomedical Optics (*n* = 36), BMC Oral Health (*n* = 36), Journal of Dental Research (*n* = 31), Photodiagnosis and Photodynamic Therapy (*n* = 31) and Oral Surgery, Oral Medicine, Oral Pathology, Oral Radiology, and Endodontics (*n* = 30) were the top 10 journals with the highest number of publications on caries diagnostic approaches (Figure 5). United States (*n* = 1092), Brazil (*n =* 698), India (*n =* 583), United Kingdom (*n =* 440), China (*n =* 435), Germany (*n =* 354), Japan (*n =* 294), Turkey (*n =* 231), Italy (*n =* 208) and Australia (*n =* 184) were the top 10 countries with the highest number of publications on caries diagnostic approaches (Figure 6). University of São Paulo (*n =* 195), State University of Campinas (*n =* 98), University of California, San Francisco (*n =* 96), University of Bern (*n =* 88), Copenhagen University (*n =* 79), Ludwig Maximilian University of Munich (*n =* 67), Federal University of Minas Gerais (*n =* 66), São Paulo State University (*n =* 64), University of Dundee (*n =* 61) and University of Michigan, Ann Arbor (*n =* 60) were the top 10 affiliations with the highest number of publications on caries diagnostic approaches (Figure 7).

The publication that had the highest citation count (CC, counted on March 31, 2024) was on visual caries detection using the International Caries Detection and Assessment System, which serves as an integrated system in classifying caries stages and activity based on the extent of caries progression and lesion activity^19^ (CC = 932) (Table 1). This was followed by a publication appertaining to the clinical application of a molecular-based diagnostic approach, which delves into the feasibility of saliva biomarkers for caries detection^20^ (CC = 554); and a publication that expatiated on the utility of deep learning-based convolutional neural network algorithm on caries diagnosis in periapical radiographs^21^ (CC = 428). Following, a publication on ICDAS that facilitates caries epidemiology and appropriate management^22^ (CC = 339), a publication on ICDAS and ICCMS for staging and managing caries^23^ (CC = 299), 3 publications on the saliva diagnostic for caries^24^ (CC = 281),^25^ (CC = 257),^26^ (CC = 240), a publication on the novel technology on caries detection^27^ (CC = 245), a publication on radiograph-based diagnostic approach^28^ (CC = 232) and a publication on laser fluorescence for caries detection^29^ (CC = 232) were also included in the top 10 publications with the highest number of citation counts.

**Table 1:** The top 10 publications on caries diagnostic approaches with the highest number of citation counts from 2003 to 2023 (By March 31, 2024).

Authors, year (Ref)	Journal	Citation count
Ismail et al. (2007)^19^	Community Dentistry and Oral Epidemiology	932
Pfaffe et al. (2011)^20^	Clinical Chemistry	554
Lee et al. (2018)^21^	Journal of Dentistry	428
Pitts (2004)^22^	Community Dental Health	339
Pitts & Ekstrand (2013)^23^	Community Dentistry and Oral Epidemiology	299
Zhang et al. (2016)^24^	International Journal of Oral Science	281
Spielmann & Wong (2011)^25^	Oral Diseases	257
Pretty (2006)^27^	Journal of Dentistry	245
Javaid et al. (2016)^26^	Journal of Oral Biology and Craniofacial Research	240
Wang et al. (2016)^28^	Medical Image Analysis	232
Baders & Shugars (2004)^29^	Journal of the American Dental Association	232

**Fig. 8:**
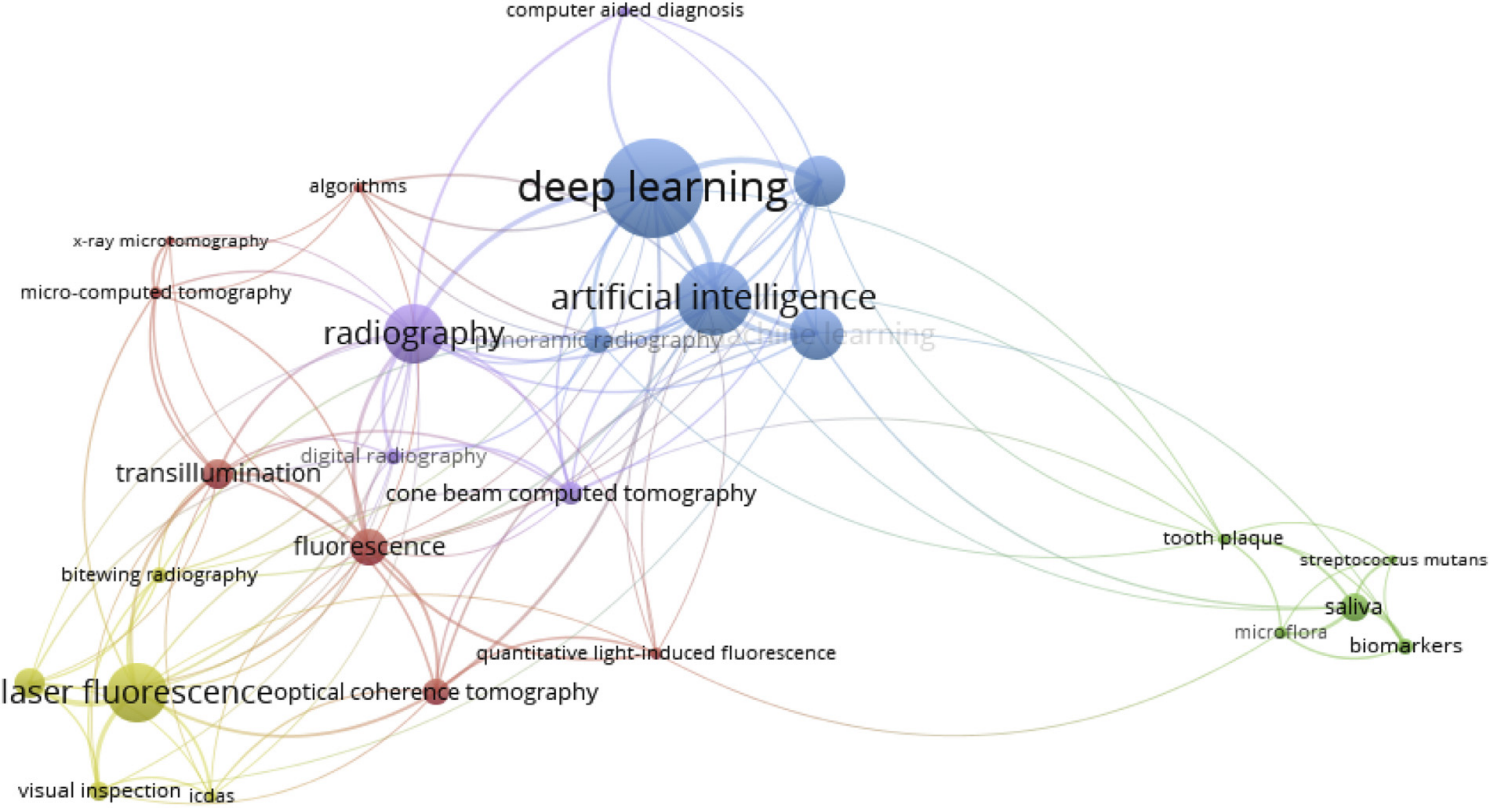
The network visualization map characterizes different clusters of keywords that occur most frequently and publications’ keyword co-occurrence link.

The network visualization map of the keywords revealed that clusters of the most frequently occurring keywords corresponded to the seven caries diagnostic approach categories (Figure 8). Several significant clusters were more prominent compared to the other clusters, including ‘Deep Learning’, ‘Artificial intelligence’, ‘Radiography’ and ‘Laser Fluorescence’.

## Discussion

This is the first bibliometric analysis of studies on caries diagnostic approaches in the last 2 decades. Throughout the past 20 years, a massive number of studies on the different diagnostic approaches for dental caries have been conducted and published. This bibliometric analysis highlighted an emerging research trend delineating different caries diagnostic approaches. This renders the importance of discerning and understanding various caries diagnostic approaches inevitable as they play an indispensable role in the diagnosis and management of dental caries.^10^

A dramatic rise in the annual number of publications on caries diagnostic approaches was observed in the past 20 years. The boom in interest in this research direction may stem from a shift of paradigm in caries diagnosis. In the past, the diagnosis of dental caries was made based on the formation of a cavity on a tooth surface, while a demineralised tooth surface with no obvious cavitation was neglected.^30^ The detection and diagnosis of cavitated caries were distinct and straightforward. Therefore, caries diagnosis was not a difficult job for clinicians in the past. More recently, a non-cavitated tooth surface with signs of demineralization was classified as early-stage caries.^16^ Diagnosing early-stage caries can be challenging and requires the aid of technology, leading to a remarkable increase in the number of studies on caries diagnostic approaches.

This bibliometric study identified seven categories of caries diagnostic approaches, including visual and/or tactile, radiation-based, light-based, ultrasound-based, electric-based and molecular-based diagnostic approaches, as well as AI-based diagnostic interpretation aids. Visual inspection with or without tactile assessment involves the use of bare eyes and dental mirrors with or without the use of a dental probe to assess the presence, severity and activity of carious lesions. It must be performed in all patients as a baseline reference.^31^ Radiograph-based diagnostic approaches refer to methods of diagnosing dental conditions and assessing oral health using radiographic images, commonly known as dental X-rays. These diagnostic approaches involve capturing images of the teeth, jawbone, soft tissue and surrounding oral structures using ionizing radiation.^28^ Light-based diagnostic caries approaches detect dental caries with the aid of various types of light and lasers. It makes use of specific wavelengths of light to assess the condition of the tooth structure and identify areas of demineralization or cavities.^18^ The ultrasound-based diagnostic approach utilizes high-frequency sound waves (ultrasound), which transmit a signal from an approximal cavitated carious lesion.^32^ The electric-based diagnostic approach measures the change of electrical current or electrical conductance resistance of a carious tooth through passing alternating current by a probe to a counter-electrode held in the patient's hand.^33^^,^^34^ Molecular-based caries diagnostic approach detects the changes of molecular biomarkers of caries in the saliva, such as enzymes, hormones, antibodies and cytokines in patients with caries.^35^^,^^36^ Regarding AI-based diagnostic interpretation aids, they are built based on convolutional neural networks and are biologically inspired programming architectures that enable computers to learn by integrating spatial information of the images and transforming data patterns into caries diagnosis.^37^

Among the publications on caries diagnostic approaches in the past 20 years, light-based caries diagnostic approaches were investigated in the highest number of studies (*n =* 771), followed by radiation-based (*n =* 662) and visual and/or tactile-based caries diagnostic approaches (*n =* 459). Common light-based diagnostic approaches include laser/light-induced fluorescence, fibre-optic/digital transillumination and optical coherence tomography.^18^ Light-based diagnostic approaches are ionizing-radiation-free, and have demonstrated good potential for the detection of caries lesions in occlusal and smooth surfaces, further establishing evidence as to why they have drawn so much attention in research in recent decades and represent the most popular ones in publications among the 7 caries diagnostic approaches.^38^ Radiation-based diagnostic approaches for caries detection commonly include bitewing X-rays, periapical X-rays, panoramic X-rays, and cone-beam computed tomography (CBCT).^39^ The immense number of publications related to radiograph-based diagnostic approaches may be attributed to its high penetration rate in clinical settings and its broad clinical application in dental practice.^31^ Visual and/or tactile-based caries diagnostic approach was another major research focus in the past 20 years and has served as the gold standard for the detection of occlusal caries. It could be due to the development and modification of detailed and validated visual index systems for caries diagnosis, such as the International Caries Detection and Assessment System (ICDAS). These visual systems allow a more accurate diagnosis of caries lesions by providing dentists with a comprehensive framework on how to characterize the identified lesions, which in turn minimizes heterogeneity and bias.^40^ Publications on ultrasound-based and electric-based caries diagnostic approaches were not as pervasive, potentially due to the complexity of applying these technologies in clinical settings in the current status.^16^^,^^41^^,^^42^

Molecular-based caries diagnostic approach has prevailed since 2016. Owing to the development of high throughput omics technologies and sophisticated molecular detection methods, the identification of minute amounts of molecules, which might act as biomarkers for dental caries detection or diagnosis, has become feasible.^43^ The potential biomarkers for caries diagnosis included microorganisms in the oral cavity, salivary proteins and peptides and salivary electrolytes.^44^ As the collection of the samples for oral biomarker detection is normally non-invasive, easily accessible and has minimal discomfort to patients, a molecular-based caries diagnostic approach could be a promising alternative to other caries diagnostic approaches. It is expected to facilitate a more personalized, valid and reliable caries diagnosis.^36^^,^^45^

A tremendous rise in the number of publications on the application of artificial intelligence (AI) as caries diagnostic interpretation aids was observed from 2020 to 2023.^46^ AI has been used in assisting caries detection based on images such as photographs and radiographs, where it is challenging to objectively identify caries lesions solely using human naked eyes.^21^ Through multiple convolutional layers with hierarchical feature representations and tooth surface segmentation, AI has demonstrated heightened proficiency in deciphering photographs and radiographs to distinguish the location and morphological changes of dental carious lesions precisely, consistently and efficiently.^47^^,^^48^ In addition, AI-based interpretation aids for caries diagnosis promote the development of teledentistry, which is a promising field using telecommunications and allied technology for the provision of remote dental care services in real time.^49^^,^^50^ AI-based interpretation aids can also assist dental professionals in diagnosing dental caries on images in teledentistry.^50^^,^^51^ Due to its profound performance in caries detection, AI-based caries diagnostic interpretation aids have become a trendy research direction in recent years.

Caries Research was the most predominant journal with the highest number of publications related to caries detection. Established in 1967, the Journal of the European Organization for Caries Research was created to advance studies centred on dental caries and other related dental diseases through epidemiological, clinical, and laboratory studies.^52^ Due to its international reputation, influence, and broad scope that encompasses the field of dental caries, this journal was frequently sought among authors to assist in clinical decision-making and conduct research on dental caries.^52^ This bibliometric analysis also found that relevant studies were often published in prestigious journals in dental sciences including Clinical Oral Investigations, BMC Oral Health and Journal of Dental Research, and journals in basic health sciences including Progress in Biomedical Optics and Imaging-Proceedings of SPIE and Journal of Biomedical Optics. All of which underscores the importance of caries detection in dentistry.

Distributions of scientific productivity can serve as an indicator of a country's research capabilities and technological advancements.^53^ The country with the highest number of publications on caries diagnostic approaches was the United States, which made significant contributions to the field of caries research.^54^ Previous studies have illustrated that the United States has been the most productive country with publications on caries detection, which might be a consequence of having high priorities in biomedical research and higher levels of funding on oral health and novel technologies.^55^ Following the USA, there has been a noticeable expansion in the number of publications in Brazil and India, which could be attributed to the sufficient manpower in research, a relatively low cost of manpower and frequent collaboration between academic institutions on an international scale.^56^

The first two among the top 10 affiliations with the highest number of publications on caries diagnostic approaches originated from the USA and Brazil, which was consistent with the aforementioned discussion on the top 10 countries. The University of São Paulo was the leading affiliation with the highest number of publications. The superior quality of Western institutions and research centres as well as the ample availability of funding for research development may cast light on this result.^57^

The number of citations is a benchmark employed to evaluate the quality of a research paper.^56^ Typically, a highly cited publication possesses the potential to significantly impact research and clinical applications.^58^ The top 10 publications that obtained the highest citation count were related to diagnostic interpretation aids, visual-based diagnostic approaches and molecular-based diagnostic approaches, which corresponded to the current research trend on caries diagnostic approaches. The considerable interest in these publications further affirmed that alternative diagnostic approaches can be used as an adjunct to reinforce the conventional visual inspection method and that the notable increase in research towards individualized AI applications is warranted to optimize caries detection.^59^^,^^60^

Keywords play a crucial role in identifying papers through bibliographic searches.^61^ The selection of appropriate keywords is fundamental to retrieving relevant references, especially considering that a myriad of papers are available on diverse topics in dentistry.^57^ In combination with visual inspection, both radiation-based and light-based caries diagnostic approaches are of paramount importance in caries detection, thus exemplifying ‘Laser Fluorescence’ and ‘Radiography’ as two of the most commonly published caries diagnostic approaches and appear as the most frequently occurring keywords.^31^ Technological advancements such as deep learning and artificial intelligence have revolutionized caries detection and treatment planning in recent years, which elucidates ‘Deep Learning’ and ‘Artificial intelligence’ as the other most frequently occurring keywords.

One limitation of this bibliometric analysis is the use of a single Scopus database. Nevertheless, it should be noted that the journals included in the Scopus database undergo annual audits to maintain a high level of quality and adherence to standards. As the largest abstract and citation database encompassing peer-reviewed scientific journals, books, and conference proceedings, the Scopus database was selected for data retrieval and analysis in this bibliometric analysis.

Although many caries diagnostic approaches have been comprehensively studied, robust conclusions cannot be drawn regarding their definite effectiveness due to the non-negligible sources of bias and heterogeneity in experimental settings of individual studies.^62^ Each caries diagnostic approach has limited indications and cannot provide an all-rounded and accurate caries diagnosis clinically in all types of tooth surfaces in the current status. This bibliometric analysis hopefully offers insights to guide future progression of caries detection, which is anticipated to be a popular interest in the field of dentistry.

## Conclusion

This bibliometric analysis identified seven categories of caries detection and diagnosis approaches from the publications in the past 20 years, including visual and/or tactile, radiation-based, light-based, ultrasound-based, electric-based, molecular-based diagnostic approaches and AI-based diagnostic interpretation aids. An increase in the annual number of publications on caries diagnostic approaches was observed in the past 20 years, revealing an emerging research trend in caries detection and diagnosis over the past two decades. In particular, publications concerning molecular-based caries diagnostic approaches and AI-based diagnostic interpretation aids have unfolded to yield impressive results in the most recent 5 years.

## Conflict of interest

The authors state that they have no conflict of interest.
